# The solid solution K_3.84_Ni_0.78_Fe_3.19_(PO_4_)_5_

**DOI:** 10.1107/S1600536814013609

**Published:** 2014-06-14

**Authors:** Nataliia Yu. Strutynska, Ivan V. Ogorodnyk, Oksana V. Livitska, Vyacheslav N. Baumer, Nikolay S. Slobodyanik

**Affiliations:** a Department of Inorganic Chemistry, Taras Shevchenko National University of Kyiv, 64/13, Volodymyrska St, 01601 Kyiv, Ukraine; b STC "Institute for Single Crystals", NAS of Ukraine, 60 Lenin Ave., 61001 Kharkiv, Ukraine

## Abstract

The title compound, tetra­potassium tetra­[nickel(II)/iron(III)] penta­kis­(orthophosphate), K_3.84_Ni_0.78_Fe_3.19_(PO_4_)_5_, has been obtained from a flux. The structure is isotypic with that of K_4_MgFe_3_(PO_4_)_5_. The three-dimensional framework is built up from (Ni/Fe)O_5_ trigonal bipyramids with a mixed Fe:Ni occupancy of 0.799 (8):0.196 (10) and isolated PO_4_ tetra­hedra, one of which is on a general position and one of which has -4.. site symmetry. Two K^+^ cations are statistically occupied and are distributed over two positions in hexa­gonally shaped channels that run parallel to [001]. One K^+^ cation [occupancy 0.73 (3)] is surrounded by nine O atoms, while the other K^+^ cation [occupancy 0.23 (3)] is surrounded by eight O atoms.

## Related literature   

The structure of isotypic K_4_MgFe_3_(PO_4_)_5_ was determined by Hidouri *et al.* (2008 ▶). For applications of iron-containing phosphates, see: Barpanda *et al.* (2012 ▶); Fisher *et al.* (2008 ▶); Huang *et al.* (2005 ▶); Shih (2003 ▶); Trad *et al.* (2010 ▶). For the different coordination polyhedra of iron in the structures of these compounds, see: Hidouri *et al.* (2002 ▶, 2003 ▶). Lajmi *et al.* (2002 ▶). For crystal-space analysis using Voronoi–Dirichlet polyhedra, see Blatov *et al.* (1995 ▶). For related compounds, see: Strutynska *et al.* (2014 ▶).

## Experimental   

### 

#### Crystal data  

K_3.84_Ni_0.78_Fe_3.19_(PO_4_)_5_
*M* *_r_* = 848.92
Tetragonal, 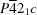

*a* = 9.6622 (6) Å
*c* = 9.380 (1) Å
*V* = 875.70 (12) Å^3^
*Z* = 2
Mo *K*α radiation
μ = 4.90 mm^−1^
*T* = 293 K
0.12 × 0.10 × 0.05 mm

#### Data collection  

Oxford Diffraction Xcalibur-3 diffractometer
Absorption correction: multi-scan (Blessing, 1995 ▶) *T*_min_ = 0.562, *T*_max_ = 0.743
14788 measured reflections
1935 independent reflections
1771 reflections with *I* > 2σ(*I*)
*R*_int_ = 0.064

#### Refinement  

*R*[*F* ^2^ > 2σ(*F* ^2^)] = 0.040
*wR*(*F* ^2^) = 0.095
*S* = 1.04
1935 reflections
82 parameters
1 restraint
Δρ_max_ = 1.02 e Å^−3^
Δρ_min_ = −1.00 e Å^−3^
Absolute structure: Flack (1983 ▶), 829 Friedel pairs
Absolute structure parameter: 0.02 (3)

### 

Data collection: *CrysAlis CCD* (Oxford Diffraction, 2006 ▶); cell refinement: *CrysAlis CCD*; data reduction: *CrysAlis RED* (Oxford Diffraction, 2006 ▶); program(s) used to solve structure: *SHELXS97* (Sheldrick, 2008 ▶); program(s) used to refine structure: *SHELXL97* (Sheldrick, 2008 ▶); molecular graphics: *DIAMOND* (Brandenburg, 1999 ▶); software used to prepare material for publication: *WinGX* (Farrugia, 2012 ▶) and *enCIFer* (Allen *et al.*, 2004 ▶).

## Supplementary Material

Crystal structure: contains datablock(s) global, I. DOI: 10.1107/S1600536814013609/wm5027sup1.cif

Structure factors: contains datablock(s) I. DOI: 10.1107/S1600536814013609/wm5027Isup2.hkl

CCDC reference: 1007841

Additional supporting information:  crystallographic information; 3D view; checkCIF report

## Figures and Tables

**Table 1:** Selected bond lengths (Å)

Fe1—O1	1.908 (3)
Fe1—O4^i^	1.908 (3)
Fe1—O3^ii^	1.918 (3)
Fe1—O5	1.975 (2)
Fe1—O2^iii^	1.979 (3)
P1—O5^iv^	1.531 (3)
P1—O5	1.531 (2)
P1—O5^v^	1.531 (3)
P1—O5^vi^	1.531 (2)
P2—O2	1.510 (3)
P2—O4	1.514 (3)
P2—O3	1.520 (3)
P2—O1	1.542 (3)

Symmetry codes: (i) 
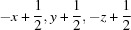
; (ii) 
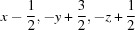
; (iii) 
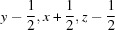
; (iv) 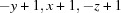; (v) 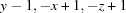; (vi) 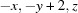.

**Fig. 1.**
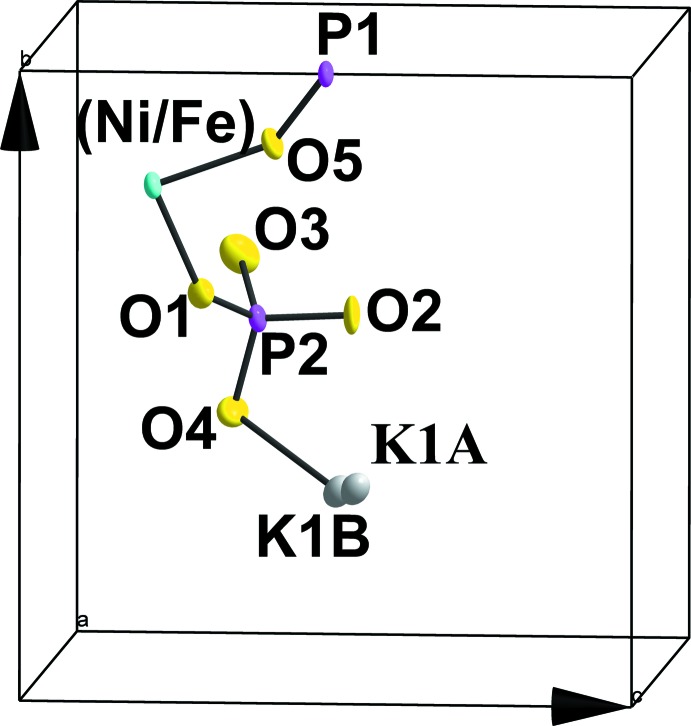
The asymmetric unit of (I), showing displacement ellipsoids at the 50% probability level.

**Fig. 2.**
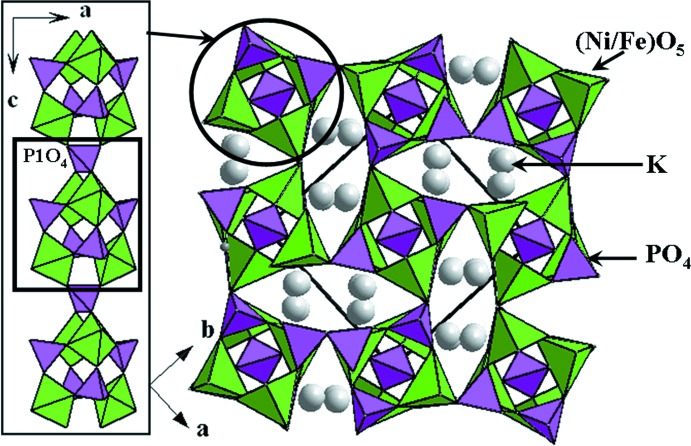
The main building blocks and their linkage into chains and the three-dimensional framework for (I) in polyhedral representation.

## supplementary crystallographic information

### S1. Comment

Complex iron-containing phosphates have different applications, for example as ionic conductors (Fisher *et al.*, 2008), cathode materials (Barpanda *et al.*, 2012; Trad *et al.*, 2010) and matrices for storage of nuclear waste (Huang *et al.*, 2005; Shih, 2003). In the crystal structures of these compounds the iron cations can adopt different coordination numbers and hence different oxygen polyhedra: FeO_4_ (Hidouri *et al.*, 2002), FeO_5_ (Hidouri *et al.*, 2003) or FeO_6_ (Lajmi *et al.*, 2002). Herein, the structure of the solid solution K_3.84_Ni_0.78_Fe_3.19_(PO_4_)_5_, tetrapotassium tetra(nickel(II)/iron(III)) pentakis(orthophosphate), (I), is reported. The crystal structure of (I) is isotypic with K_4_MgFe_3_(PO_4_)_5_ (Hidouri *et al.*, 2008).

The asymmetric unit of (I) consists of one mixed-occupied (Ni^II^/Fe^III^) site, two P sites (one of which is located on a fourfold rotoinversion axis), five oxygen sites and two K^+^ sites which are partly occupied and distributed over two positions (K1A and K1B) (Fig. 1). The main building blocks are one [(Ni/Fe^III^)O_5_] trigonal bipyramid and two [PO_4_] tetrahedra. The [(Ni/Fe^III^)O_5_] polyhedron is linked with [P1O_4_] tetrahedra into chains along [001] which additionally are aggregated by the linkage with [P2O_4_] tetrahedra into a three-dimensional framework with composition [Ni_0.78_Fe_3.19_(PO_4_)_5_]^3.84-^ (Fig. 2).

The environment of the mixed (Ni^II^/Fe^III^) site is defined by five oxygen atoms from four [P2O_4_] tetrahedra and one [P1O_4_] tetrahedron. The distances (Ni/Fe)—O vary between 1.908 (3) and 1.979 (3) Å. The average distance ((Ni/Fe)—O) = 1.937 Å is slightly less than that in K_4_MgFe_3_(PO_4_)_5_ (*d*((Mg/Fe)—O) = 1.952 Å) (Hidouri *et al.*, 2008). The tetrahedral orthophosphate anions deviate only slightly from ideal values with P—O bond lengths ranging from 1.510 (3) to 1.542 (3) Å.

The disordered K^+^ cations are located in hexagonally-shaped channels running along [001], with occupancies of 0.73 (3) (K1A) and 0.23 (3) (K1B). The results of the construction of Voronoi-Dirichlet polyhedra (Blatov *et al.*, 1995) show the K1A being surrounded by nine O atoms while K1B is surrounded by eight O atoms. The K—O distances in the [K1AO_9_]-polyhedron are in the range 2.719 (5)–3.072 (6) Å, while in the [K1BO_8_]-polyhedron they are in the range 2.636 (13)–3.065 (15) Å.

The main difference between the obtained solid solution and the phosphate K_4_MgFe_3_(PO_4_)_5_ (Hidouri *et al.*, 2008) is the splitting of the K^+^ site in two positions. The occupation of the K1B site (0.23 (3)) correlates with the increase of the iron content (from 3 to 3.19) in the starting matrix [*M*^II^Fe^III^_3_(PO_4_)_5_]^4-^. It seems that a partial substitution of Ni by Fe in [*M*^II^Fe^III^_3_(PO_4_)_5_]^4-^ causes the formation of vacancies in the cationic K^+^ lattice and a splitting of the respective K^+^ site. A similar influence of an heterovalent substitution on the splitting of alkaline metal sites was found for KNi_0.93_Fe^II^_0.07_Fe^III^(PO_4_)_2_ (Strutynska *et al.*, 2014).

### S2. Experimental

The title compound was obtained during investigation of the melting system K_2_O–P_2_O_5_–Fe_2_O_3_–NiO–MoO_3_. A mixture of KPO_3_ (14.16 g), NiO (2.70 g), Fe_2_O_3_ (2.88 g) and K_2_Mo_2_O_7_ (4 g) was ground in an agate mortar, placed in a platinum crucible and heated up to 1273 K. The melt was kept at this temperature for 3 h. After that, the temperature was cooled down to 873 K at a rate of 10 K/h. The crystals of (I) were separated from the remaining flux by boiling with water. The chemical composition of selected single-crystal was verified by EDX analysis. Analysis found (calculated) for K_3.84_Ni_0.78_Fe_3.19_ (PO_4_)_5_ in atomic percentage: K 17.62 (17.69), Ni 5.34 (5.39), Fe 20.83 (20.99), P 18.44 (18.24) and O 37.77 (37.69).

### S3. Refinement

Because of the similarity of possible coordination by O atoms, Ni and Fe were placed on the same site. Their coordinates and anisotropic displacement parameters (ADP) were constrained to be equal. The corresponding occupancy factors were refined using free variables. After that procedure, an unidentified high electron density peak was found near the position of the K site. It was supposed that this site can be occupied only by another K^+^ caion. ADPs of both split K sites were constrained to be equal, while the occupancies were refined using free variables. The calculated occupancy factors of all partially occupied positions were close to those reported in this paper. To fix the electroneutrality of the compound, SUMP restraints in SHELXL (Sheldrick, 2008) were applied to the occupancy factors of the refined atoms.

The highest and lowest electron densities were found 1.00 Å from O1 and 0.76 Å from NI1, respectively.

### Crystal data

**Table:**

K_3.84_Ni_0.78_Fe_3.19_(PO_4_)_5_	*D*_x_ = 3.222 Mg m^−^^3^
*M**_r_* = 848.92	Mo *K*α radiation, λ = 0.71073 Å
Tetragonal, *P*42_1_*c*	Cell parameters from 14788 reflections
Hall symbol: P -4 2 n	θ = 3.0–35°
*a* = 9.6622 (6) Å	µ = 4.90 mm^−^^1^
*c* = 9.380 (1) Å	*T* = 293 K
*V* = 875.70 (12) Å^3^	Prism, yellow
*Z* = 2	0.12 × 0.10 × 0.05 mm
*F*(000) = 826	

### Data collection

**Table:**

Oxford Diffraction Xcalibur-3 diffractometer	1935 independent reflections
Radiation source: fine-focus sealed tube	1771 reflections with *I* > 2σ(*I*)
Graphite monochromator	*R*_int_ = 0.064
φ and ω scans	θ_max_ = 35°, θ_min_ = 3.0°
Absorption correction: multi-scan (Blessing, 1995)	*h* = −15→15
*T*_min_ = 0.562, *T*_max_ = 0.743	*k* = −15→15
14788 measured reflections	*l* = −15→15

### Refinement

**Table:**

Refinement on *F*^2^	Primary atom site location: structure-invariant direct methods
Least-squares matrix: full	Secondary atom site location: difference Fourier map
*R*[*F*^2^ > 2σ(*F*^2^)] = 0.040	*w* = 1/[σ^2^(*F*_o_^2^) + (0.050*P*)^2^ + 0.8951*P*] where *P* = (*F*_o_^2^ + 2*F*_c_^2^)/3
*wR*(*F*^2^) = 0.095	(Δ/σ)_max_ < 0.001
*S* = 1.04	Δρ_max_ = 1.02 e Å^−^^3^
1935 reflections	Δρ_min_ = −1.00 e Å^−^^3^
82 parameters	Absolute structure: Flack (1983), 829 Friedel pairs
1 restraint	Absolute structure parameter: 0.02 (3)

### Special details

**Table:**

Geometry. All e.s.d.'s (except the e.s.d. in the dihedral angle between two l.s. planes) are estimated using the full covariance matrix. The cell e.s.d.'s are taken into account individually in the estimation of e.s.d.'s in distances, angles and torsion angles; correlations between e.s.d.'s in cell parameters are only used when they are defined by crystal symmetry. An approximate (isotropic) treatment of cell e.s.d.'s is used for estimating e.s.d.'s involving l.s. planes.
Refinement. Refinement of *F*^2^ against ALL reflections. The weighted *R*-factor *wR* and goodness of fit *S* are based on *F*^2^, conventional *R*-factors *R* are based on *F*, with *F* set to zero for negative *F*^2^. The threshold expression of *F*^2^ > σ(*F*^2^) is used only for calculating *R*-factors(gt) *etc*. and is not relevant to the choice of reflections for refinement. *R*-factors based on *F*^2^ are statistically about twice as large as those based on *F*, and *R*- factors based on ALL data will be even larger.

### Fractional atomic coordinates and isotropic or equivalent isotropic displacement parameters (Å^2^)

**Table:**

	*x*	*y*	*z*	*U*_iso_*/*U*_eq_	Occ. (<1)
Fe1	0.07474 (5)	0.81210 (5)	0.21055 (5)	0.01399 (11)	0.799 (8)
Ni1	0.07474 (5)	0.81210 (5)	0.21055 (5)	0.01399 (11)	0.196 (10)
K1A	0.0677 (6)	0.3344 (4)	0.5415 (10)	0.0267 (8)	0.73 (3)
K1B	0.0837 (15)	0.3284 (14)	0.5131 (17)	0.0267 (8)	0.23 (3)
P1	0	1	0.5	0.0138 (3)	
P2	0.25560 (9)	0.58266 (10)	0.36473 (8)	0.01535 (18)	
O1	0.1268 (3)	0.6356 (3)	0.2843 (3)	0.0238 (5)	
O2	0.2226 (3)	0.5930 (3)	0.5217 (3)	0.0245 (6)	
O3	0.3798 (4)	0.6706 (4)	0.3236 (4)	0.0361 (8)	
O4	0.2718 (3)	0.4322 (3)	0.3226 (3)	0.0289 (6)	
O5	0.0560 (3)	0.8822 (3)	0.4074 (3)	0.0204 (5)	

### Atomic displacement parameters (Å^2^)

**Table:**

	*U*^11^	*U*^22^	*U*^33^	*U*^12^	*U*^13^	*U*^23^
Fe1	0.0157 (2)	0.0181 (2)	0.00817 (16)	0.00085 (16)	−0.00043 (16)	−0.00122 (15)
Ni1	0.0157 (2)	0.0181 (2)	0.00817 (16)	0.00085 (16)	−0.00043 (16)	−0.00122 (15)
K1A	0.0289 (10)	0.0276 (6)	0.0235 (18)	−0.0078 (6)	−0.0031 (11)	0.0056 (9)
K1B	0.0289 (10)	0.0276 (6)	0.0235 (18)	−0.0078 (6)	−0.0031 (11)	0.0056 (9)
P1	0.0177 (5)	0.0177 (5)	0.0061 (6)	0	0	0
P2	0.0180 (4)	0.0198 (4)	0.0083 (3)	−0.0018 (3)	0.0008 (3)	−0.0018 (3)
O1	0.0294 (13)	0.0228 (12)	0.0191 (11)	0.0015 (10)	−0.0095 (11)	0.0004 (10)
O2	0.0299 (13)	0.0363 (15)	0.0074 (9)	0.0067 (13)	−0.0033 (8)	0.0001 (10)
O3	0.0270 (14)	0.0427 (19)	0.0385 (18)	−0.0108 (14)	0.0101 (13)	−0.0073 (15)
O4	0.0402 (16)	0.0222 (12)	0.0243 (13)	0.0091 (12)	0.0056 (11)	−0.0014 (11)
O5	0.0245 (13)	0.0247 (12)	0.0120 (9)	0.0010 (10)	0.0020 (9)	−0.0054 (9)

### Geometric parameters (Å, º)

**Table:**

Fe1—O1	1.908 (3)	P1—K1B^xii^	3.277 (13)
Fe1—O4^i^	1.908 (3)	P1—K1B^iv^	3.277 (13)
Fe1—O3^ii^	1.918 (3)	P1—K1B^xiii^	3.277 (13)
Fe1—O5	1.975 (2)	P1—K1B^v^	3.277 (13)
Fe1—O2^iii^	1.979 (3)	P1—K1A^iv^	3.319 (4)
Fe1—K1B^iv^	3.498 (14)	P1—K1A^xii^	3.319 (4)
Fe1—K1A^v^	3.613 (4)	P1—K1A^xiii^	3.319 (4)
Fe1—K1A^iii^	3.672 (4)	P1—K1A^v^	3.319 (4)
Fe1—K1A^iv^	3.679 (7)	P2—O2	1.510 (3)
Fe1—K1B^v^	3.707 (13)	P2—O4	1.514 (3)
Fe1—K1B^iii^	3.737 (13)	P2—O3	1.520 (3)
Fe1—K1B^i^	3.914 (17)	P2—O1	1.542 (3)
K1A—O5^iv^	2.719 (5)	P2—K1A^xiv^	3.473 (9)
K1A—O5^vi^	2.774 (5)	P2—K1B^v^	3.493 (13)
K1A—O4^vii^	2.830 (8)	P2—K1A^v^	3.573 (4)
K1A—O3^vi^	2.862 (5)	P2—K1A^iv^	3.626 (3)
K1A—O2^iv^	2.898 (6)	P2—K1B^iv^	3.664 (13)
K1A—O2	2.919 (5)	P2—K1B^xiv^	3.758 (16)
K1A—O4	3.000 (9)	O1—K1B^iv^	2.978 (13)
K1A—O1^vii^	3.031 (10)	O1—K1A^xiv^	3.031 (10)
K1A—O1^iv^	3.072 (6)	O1—K1A^iv^	3.072 (6)
K1A—P1^viii^	3.319 (4)	O1—K1B^xiv^	3.339 (18)
K1A—P2	3.435 (6)	O2—Ni1^xv^	1.979 (3)
K1A—K1A^iv^	3.458 (10)	O2—Fe1^xv^	1.979 (3)
K1B—O5^iv^	2.636 (13)	O2—K1A^iv^	2.898 (6)
K1B—O4	2.739 (16)	O2—K1B^iv^	3.057 (15)
K1B—O5^vi^	2.755 (13)	O2—K1B^v^	3.303 (16)
K1B—O3^vi^	2.869 (13)	O3—Ni1^xvi^	1.918 (3)
K1B—O2	2.889 (13)	O3—Fe1^xvi^	1.918 (3)
K1B—O1^iv^	2.978 (13)	O3—K1A^v^	2.862 (5)
K1B—O2^iv^	3.057 (15)	O3—K1B^v^	2.869 (13)
K1B—O4^vii^	3.065 (15)	O4—Ni1^xvii^	1.908 (3)
K1B—P2	3.276 (13)	O4—Fe1^xvii^	1.908 (3)
K1B—P1^viii^	3.277 (13)	O4—K1A^xiv^	2.830 (8)
K1B—O2^vi^	3.303 (16)	O4—K1B^xiv^	3.065 (15)
K1B—O1^vii^	3.339 (18)	O5—K1B^iv^	2.636 (13)
P1—O5^ix^	1.531 (3)	O5—K1A^iv^	2.719 (5)
P1—O5	1.531 (2)	O5—K1B^v^	2.755 (13)
P1—O5^x^	1.531 (3)	O5—K1A^v^	2.774 (5)
P1—O5^xi^	1.531 (2)		
			
O1—Fe1—O4^i^	113.45 (13)	O5^x^—P1—K1B^iv^	56.8 (2)
O1—Fe1—O3^ii^	113.43 (14)	O5^xi^—P1—K1B^iv^	130.8 (3)
O4^i^—Fe1—O3^ii^	133.04 (15)	K1B^xii^—P1—K1B^iv^	175.7 (5)
O1—Fe1—O5	89.54 (12)	O5^ix^—P1—K1B^xiii^	130.8 (3)
O4^i^—Fe1—O5	90.85 (12)	O5—P1—K1B^xiii^	120.3 (3)
O3^ii^—Fe1—O5	92.08 (13)	O5^x^—P1—K1B^xiii^	52.3 (2)
O1—Fe1—O2^iii^	84.85 (12)	O5^xi^—P1—K1B^xiii^	56.8 (2)
O4^i^—Fe1—O2^iii^	92.89 (13)	K1B^xii^—P1—K1B^xiii^	90.08 (2)
O3^ii^—Fe1—O2^iii^	88.65 (13)	K1B^iv^—P1—K1B^xiii^	90.08 (2)
O5—Fe1—O2^iii^	174.16 (12)	O5^ix^—P1—K1B^v^	52.3 (2)
O1—Fe1—K1B^iv^	58.3 (3)	O5—P1—K1B^v^	56.8 (2)
O4^i^—Fe1—K1B^iv^	135.1 (3)	O5^x^—P1—K1B^v^	130.8 (3)
O3^ii^—Fe1—K1B^iv^	74.9 (3)	O5^xi^—P1—K1B^v^	120.3 (3)
O5—Fe1—K1B^iv^	48.3 (2)	K1B^xii^—P1—K1B^v^	90.08 (2)
O2^iii^—Fe1—K1B^iv^	126.6 (2)	K1B^iv^—P1—K1B^v^	90.08 (2)
O1—Fe1—K1A^v^	82.58 (14)	K1B^xiii^—P1—K1B^v^	175.7 (5)
O4^i^—Fe1—K1A^v^	50.96 (18)	O5^ix^—P1—K1A^iv^	115.1 (2)
O3^ii^—Fe1—K1A^v^	139.59 (13)	O5—P1—K1A^iv^	54.03 (13)
O5—Fe1—K1A^v^	49.57 (14)	O5^x^—P1—K1A^iv^	56.12 (12)
O2^iii^—Fe1—K1A^v^	130.90 (13)	O5^xi^—P1—K1A^iv^	136.1 (2)
K1B^iv^—Fe1—K1A^v^	84.63 (17)	K1B^xii^—P1—K1A^iv^	170.7 (4)
O1—Fe1—K1A^iii^	75.43 (16)	K1B^iv^—P1—K1A^iv^	5.40 (18)
O4^i^—Fe1—K1A^iii^	144.29 (15)	K1B^xiii^—P1—K1A^iv^	93.1 (3)
O3^ii^—Fe1—K1A^iii^	50.43 (13)	K1B^v^—P1—K1A^iv^	87.4 (3)
O5—Fe1—K1A^iii^	124.50 (14)	O5^ix^—P1—K1A^xii^	56.12 (12)
O2^iii^—Fe1—K1A^iii^	52.34 (13)	O5—P1—K1A^xii^	136.1 (2)
K1B^iv^—Fe1—K1A^iii^	79.86 (19)	O5^x^—P1—K1A^xii^	115.1 (2)
K1A^v^—Fe1—K1A^iii^	157.50 (3)	O5^xi^—P1—K1A^xii^	54.03 (13)
O1—Fe1—K1A^iv^	56.57 (12)	K1B^xii^—P1—K1A^xii^	5.40 (18)
O4^i^—Fe1—K1A^iv^	131.48 (15)	K1B^iv^—P1—K1A^xii^	170.7 (4)
O3^ii^—Fe1—K1A^iv^	78.87 (17)	K1B^xiii^—P1—K1A^xii^	87.4 (3)
O5—Fe1—K1A^iv^	46.28 (11)	K1B^v^—P1—K1A^xii^	93.1 (3)
O2^iii^—Fe1—K1A^iv^	128.36 (11)	K1A^iv^—P1—K1A^xii^	166.5 (3)
K1B^iv^—Fe1—K1A^iv^	4.1 (2)	O5^ix^—P1—K1A^xiii^	136.1 (2)
K1A^v^—Fe1—K1A^iv^	80.81 (6)	O5—P1—K1A^xiii^	115.1 (2)
K1A^iii^—Fe1—K1A^iv^	83.13 (5)	O5^x^—P1—K1A^xiii^	54.03 (13)
O1—Fe1—K1B^v^	79.3 (2)	O5^xi^—P1—K1A^xiii^	56.12 (12)
O4^i^—Fe1—K1B^v^	55.6 (3)	K1B^xii^—P1—K1A^xiii^	93.1 (3)
O3^ii^—Fe1—K1B^v^	137.9 (2)	K1B^iv^—P1—K1A^xiii^	87.4 (3)
O5—Fe1—K1B^v^	46.6 (2)	K1B^xiii^—P1—K1A^xiii^	5.40 (18)
O2^iii^—Fe1—K1B^v^	133.3 (2)	K1B^v^—P1—K1A^xiii^	170.7 (4)
K1B^iv^—Fe1—K1B^v^	80.1 (2)	K1A^iv^—P1—K1A^xiii^	90.79 (4)
K1A^v^—Fe1—K1B^v^	4.68 (18)	K1A^xii^—P1—K1A^xiii^	90.79 (4)
K1A^iii^—Fe1—K1B^v^	153.49 (18)	O5^ix^—P1—K1A^v^	54.03 (13)
K1A^iv^—Fe1—K1B^v^	76.22 (18)	O5—P1—K1A^v^	56.12 (12)
O1—Fe1—K1B^iii^	79.6 (3)	O5^x^—P1—K1A^v^	136.1 (2)
O4^i^—Fe1—K1B^iii^	140.6 (2)	O5^xi^—P1—K1A^v^	115.1 (2)
O3^ii^—Fe1—K1B^iii^	49.0 (2)	K1B^xii^—P1—K1A^v^	87.4 (3)
O5—Fe1—K1B^iii^	127.4 (2)	K1B^iv^—P1—K1A^v^	93.1 (3)
O2^iii^—Fe1—K1B^iii^	49.9 (2)	K1B^xiii^—P1—K1A^v^	170.7 (4)
K1B^iv^—Fe1—K1B^iii^	83.93 (9)	K1B^v^—P1—K1A^v^	5.40 (18)
K1A^v^—Fe1—K1B^iii^	162.0 (2)	K1A^iv^—P1—K1A^v^	90.79 (4)
K1A^iii^—Fe1—K1B^iii^	4.74 (16)	K1A^xii^—P1—K1A^v^	90.79 (4)
K1A^iv^—Fe1—K1B^iii^	87.29 (19)	K1A^xiii^—P1—K1A^v^	166.5 (3)
K1B^v^—Fe1—K1B^iii^	158.14 (6)	O2—P2—O4	109.86 (17)
O1—Fe1—K1B^i^	90.4 (2)	O2—P2—O3	112.16 (19)
O4^i^—Fe1—K1B^i^	39.9 (2)	O4—P2—O3	112.85 (18)
O3^ii^—Fe1—K1B^i^	137.3 (2)	O2—P2—O1	106.56 (17)
O5—Fe1—K1B^i^	124.4 (2)	O4—P2—O1	105.92 (17)
O2^iii^—Fe1—K1B^i^	57.5 (2)	O3—P2—O1	109.10 (19)
K1B^iv^—Fe1—K1B^i^	145.0 (3)	O2—P2—K1B	61.9 (3)
K1A^v^—Fe1—K1B^i^	75.32 (18)	O4—P2—K1B	56.2 (3)
K1A^iii^—Fe1—K1B^i^	109.1 (2)	O3—P2—K1B	158.0 (3)
K1A^iv^—Fe1—K1B^i^	141.56 (17)	O1—P2—K1B	92.7 (3)
K1B^v^—Fe1—K1B^i^	78.8 (2)	O2—P2—K1A	57.59 (18)
K1B^iii^—Fe1—K1B^i^	107.17 (6)	O4—P2—K1A	60.68 (19)
O5^iv^—K1A—O5^vi^	53.88 (13)	O3—P2—K1A	158.44 (17)
O5^iv^—K1A—O4^vii^	98.74 (17)	O1—P2—K1A	92.38 (12)
O5^vi^—K1A—O4^vii^	59.16 (13)	K1B—P2—K1A	4.6 (2)
O5^iv^—K1A—O3^vi^	135.3 (2)	O2—P2—K1A^xiv^	145.72 (14)
O5^vi^—K1A—O3^vi^	85.30 (13)	O4—P2—K1A^xiv^	52.89 (13)
O4^vii^—K1A—O3^vi^	69.10 (15)	O3—P2—K1A^xiv^	102.11 (15)
O5^iv^—K1A—O2^iv^	74.39 (12)	O1—P2—K1A^xiv^	60.63 (14)
O5^vi^—K1A—O2^iv^	114.49 (17)	K1B—P2—K1A^xiv^	86.0 (3)
O4^vii^—K1A—O2^iv^	98.5 (3)	K1A—P2—K1A^xiv^	89.82 (9)
O3^vi^—K1A—O2^iv^	147.9 (3)	O2—P2—K1B^v^	70.2 (3)
O5^iv^—K1A—O2	148.8 (4)	O4—P2—K1B^v^	161.9 (3)
O5^vi^—K1A—O2	138.7 (2)	O3—P2—K1B^v^	53.7 (3)
O4^vii^—K1A—O2	111.7 (2)	O1—P2—K1B^v^	91.0 (2)
O3^vi^—K1A—O2	56.21 (11)	K1B—P2—K1B^v^	130.91 (4)
O2^iv^—K1A—O2	106.56 (15)	K1A—P2—K1B^v^	126.3 (2)
O5^iv^—K1A—O4	102.4 (3)	K1A^xiv^—P2—K1B^v^	136.3 (3)
O5^vi^—K1A—O4	108.0 (3)	O2—P2—K1A^v^	75.1 (2)
O4^vii^—K1A—O4	138.76 (16)	O4—P2—K1A^v^	161.74 (15)
O3^vi^—K1A—O4	70.94 (17)	O3—P2—K1A^v^	50.65 (17)
O2^iv^—K1A—O4	121.09 (19)	O1—P2—K1A^v^	88.79 (14)
O2—K1A—O4	49.42 (13)	K1B—P2—K1A^v^	135.4 (2)
O5^iv^—K1A—O1^vii^	99.1 (2)	K1A—P2—K1A^v^	130.85 (3)
O5^vi^—K1A—O1^vii^	95.9 (2)	K1A^xiv^—P2—K1A^v^	131.60 (16)
O4^vii^—K1A—O1^vii^	49.06 (18)	K1B^v^—P2—K1A^v^	4.92 (17)
O3^vi^—K1A—O1^vii^	102.8 (3)	O2—P2—K1A^iv^	50.0 (2)
O2^iv^—K1A—O1^vii^	52.46 (16)	O4—P2—K1A^iv^	114.87 (14)
O2—K1A—O1^vii^	105.9 (2)	O3—P2—K1A^iv^	132.28 (15)
O4—K1A—O1^vii^	154.35 (18)	O1—P2—K1A^iv^	57.0 (2)
O5^iv^—K1A—O1^iv^	55.96 (12)	K1B—P2—K1A^iv^	62.3 (3)
O5^vi^—K1A—O1^iv^	109.51 (18)	K1A—P2—K1A^iv^	58.56 (19)
O4^vii^—K1A—O1^iv^	140.0 (2)	K1A^xiv^—P2—K1A^iv^	106.02 (10)
O3^vi^—K1A—O1^iv^	150.9 (3)	K1B^v^—P2—K1A^iv^	79.60 (18)
O2^iv^—K1A—O1^iv^	48.28 (9)	K1A^v^—P2—K1A^iv^	82.07 (11)
O2—K1A—O1^iv^	100.54 (17)	O2—P2—K1B^iv^	54.9 (3)
O4—K1A—O1^iv^	80.5 (2)	O4—P2—K1B^iv^	114.6 (2)
O1^vii^—K1A—O1^iv^	100.48 (17)	O3—P2—K1B^iv^	132.2 (2)
O5^iv^—K1A—P1^viii^	27.11 (6)	O1—P2—K1B^iv^	52.1 (3)
O5^vi^—K1A—P1^viii^	27.26 (6)	K1B—P2—K1B^iv^	64.0 (5)
O4^vii^—K1A—P1^viii^	75.89 (12)	K1A—P2—K1B^iv^	60.5 (3)
O3^vi^—K1A—P1^viii^	112.04 (14)	K1A^xiv^—P2—K1B^iv^	101.87 (18)
O2^iv^—K1A—P1^viii^	92.13 (12)	K1B^v^—P2—K1B^iv^	80.7 (4)
O2—K1A—P1^viii^	158.0 (3)	K1A^v^—P2—K1B^iv^	82.84 (15)
O4—K1A—P1^viii^	110.9 (3)	K1A^iv^—P2—K1B^iv^	4.89 (16)
O1^vii^—K1A—P1^viii^	94.63 (17)	O2—P2—K1B^xiv^	147.0 (2)
O1^iv^—K1A—P1^viii^	83.06 (12)	O4—P2—K1B^xiv^	51.9 (2)
O5^iv^—K1A—P2	123.1 (3)	O3—P2—K1B^xiv^	100.8 (2)
O5^vi^—K1A—P2	131.9 (3)	O1—P2—K1B^xiv^	62.5 (2)
O4^vii^—K1A—P2	134.67 (15)	K1B—P2—K1B^xiv^	86.7 (2)
O3^vi^—K1A—P2	68.87 (13)	K1A—P2—K1B^xiv^	90.6 (2)
O2^iv^—K1A—P2	108.16 (12)	K1A^xiv^—P2—K1B^xiv^	2.1 (2)
O2—K1A—P2	25.89 (8)	K1B^v^—P2—K1B^xiv^	136.6 (3)
O4—K1A—P2	26.12 (7)	K1A^v^—P2—K1B^xiv^	131.8 (3)
O1^vii^—K1A—P2	128.24 (19)	K1A^iv^—P2—K1B^xiv^	108.05 (18)
O1^iv^—K1A—P2	83.01 (17)	K1B^iv^—P2—K1B^xiv^	103.9 (3)
P1^viii^—K1A—P2	136.7 (3)	P2—O1—Fe1	133.35 (16)
O5^iv^—K1A—K1A^iv^	123.1 (2)	P2—O1—K1B^iv^	103.7 (4)
O5^vi^—K1A—K1A^iv^	164.4 (3)	Fe1—O1—K1B^iv^	88.7 (3)
O4^vii^—K1A—K1A^iv^	109.62 (17)	P2—O1—K1A^xiv^	93.04 (15)
O3^vi^—K1A—K1A^iv^	101.10 (15)	Fe1—O1—K1A^xiv^	109.87 (14)
O2^iv^—K1A—K1A^iv^	53.81 (18)	K1B^iv^—O1—K1A^xiv^	134.6 (2)
O2—K1A—K1A^iv^	53.25 (10)	P2—O1—K1A^iv^	98.1 (2)
O4—K1A—K1A^iv^	87.55 (11)	Fe1—O1—K1A^iv^	92.22 (17)
O1^vii^—K1A—K1A^iv^	68.91 (12)	K1B^iv^—O1—K1A^iv^	5.7 (2)
O1^iv^—K1A—K1A^iv^	71.40 (18)	K1A^xiv^—O1—K1A^iv^	136.63 (15)
P1^viii^—K1A—K1A^iv^	145.7 (2)	P2—O1—K1B^xiv^	93.3 (3)
P2—K1A—K1A^iv^	63.49 (9)	Fe1—O1—K1B^xiv^	109.0 (2)
O5^iv^—K1B—O4	112.2 (6)	K1B^iv^—O1—K1B^xiv^	135.4 (4)
O5^iv^—K1B—O5^vi^	54.9 (3)	K1A^xiv^—O1—K1B^xiv^	1.0 (3)
O4—K1B—O5^vi^	116.6 (6)	K1A^iv^—O1—K1B^xiv^	137.52 (19)
O5^iv^—K1B—O3^vi^	139.3 (5)	P2—O2—Ni1^xv^	140.79 (19)
O4—K1B—O3^vi^	74.7 (4)	P2—O2—Fe1^xv^	140.79 (19)
O5^vi^—K1B—O3^vi^	85.5 (4)	Ni1^xv^—O2—Fe1^xv^	0.00 (3)
O5^iv^—K1B—O2	158.7 (6)	P2—O2—K1B	90.7 (4)
O4—K1B—O2	52.1 (3)	Ni1^xv^—O2—K1B	98.6 (3)
O5^vi^—K1B—O2	141.5 (5)	Fe1^xv^—O2—K1B	98.6 (3)
O3^vi^—K1B—O2	56.5 (3)	P2—O2—K1A^iv^	106.4 (2)
O5^iv^—K1B—O1^iv^	57.9 (3)	Ni1^xv^—O2—K1A^iv^	112.8 (2)
O4—K1B—O1^iv^	86.6 (4)	Fe1^xv^—O2—K1A^iv^	112.8 (2)
O5^vi^—K1B—O1^iv^	112.9 (4)	K1B—O2—K1A^iv^	76.5 (3)
O3^vi^—K1B—O1^iv^	158.5 (5)	P2—O2—K1A	96.5 (2)
O2—K1B—O1^iv^	103.5 (4)	Ni1^xv^—O2—K1A	95.20 (17)
O5^iv^—K1B—O2^iv^	72.9 (3)	Fe1^xv^—O2—K1A	95.20 (17)
O4—K1B—O2^iv^	124.7 (5)	K1B—O2—K1A	6.2 (2)
O5^vi^—K1B—O2^iv^	110.2 (5)	K1A^iv^—O2—K1A	72.95 (18)
O3^vi^—K1B—O2^iv^	138.1 (6)	P2—O2—K1B^iv^	101.2 (3)
O2—K1B—O2^iv^	103.3 (4)	Ni1^xv^—O2—K1B^iv^	118.0 (3)
O1^iv^—K1B—O2^iv^	47.8 (2)	Fe1^xv^—O2—K1B^iv^	118.0 (3)
O5^iv^—K1B—O4^vii^	95.0 (4)	K1B—O2—K1B^iv^	76.7 (4)
O4—K1B—O4^vii^	140.1 (5)	K1A^iv^—O2—K1B^iv^	5.2 (2)
O5^vi^—K1B—O4^vii^	56.5 (3)	K1A—O2—K1B^iv^	73.6 (3)
O3^vi^—K1B—O4^vii^	65.8 (3)	P2—O2—K1B^v^	84.3 (3)
O2—K1B—O4^vii^	106.1 (4)	Ni1^xv^—O2—K1B^v^	92.2 (3)
O1^iv^—K1B—O4^vii^	133.3 (5)	Fe1^xv^—O2—K1B^v^	92.2 (3)
O2^iv^—K1B—O4^vii^	90.3 (5)	K1B—O2—K1B^v^	168.0 (3)
O5^iv^—K1B—P2	132.8 (6)	K1A^iv^—O2—K1B^v^	94.42 (19)
O4—K1B—P2	27.35 (14)	K1A—O2—K1B^v^	167.1 (3)
O5^vi^—K1B—P2	140.4 (6)	K1B^iv^—O2—K1B^v^	93.6 (5)
O3^vi^—K1B—P2	71.2 (3)	P2—O3—Ni1^xvi^	150.1 (3)
O2—K1B—P2	27.44 (13)	P2—O3—Fe1^xvi^	150.1 (3)
O1^iv^—K1B—P2	87.3 (3)	Ni1^xvi^—O3—Fe1^xvi^	0.00 (3)
O2^iv^—K1B—P2	108.4 (4)	P2—O3—K1A^v^	105.1 (2)
O4^vii^—K1B—P2	131.7 (4)	Ni1^xvi^—O3—K1A^v^	98.46 (16)
O5^iv^—K1B—P1^viii^	27.36 (14)	Fe1^xvi^—O3—K1A^v^	98.46 (16)
O4—K1B—P1^viii^	119.6 (6)	P2—O3—K1B^v^	101.0 (4)
O5^vi^—K1B—P1^viii^	27.71 (13)	Ni1^xvi^—O3—K1B^v^	100.8 (3)
O3^vi^—K1B—P1^viii^	113.1 (4)	Fe1^xvi^—O3—K1B^v^	100.8 (3)
O2—K1B—P1^viii^	166.6 (6)	K1A^v^—O3—K1B^v^	6.3 (2)
O1^iv^—K1B—P1^viii^	85.3 (3)	P2—O4—Ni1^xvii^	135.0 (2)
O2^iv^—K1B—P1^viii^	90.1 (3)	P2—O4—Fe1^xvii^	135.0 (2)
O4^vii^—K1B—P1^viii^	73.6 (3)	Ni1^xvii^—O4—Fe1^xvii^	0.00 (3)
P2—K1B—P1^viii^	146.7 (6)	P2—O4—K1B	96.5 (3)
O5^iv^—K1B—O2^vi^	102.0 (5)	Ni1^xvii^—O4—K1B	113.5 (3)
O4—K1B—O2^vi^	54.6 (3)	Fe1^xvii^—O4—K1B	113.5 (3)
O5^vi^—K1B—O2^vi^	67.5 (3)	P2—O4—K1A^xiv^	101.84 (19)
O3^vi^—K1B—O2^vi^	47.4 (2)	Ni1^xvii^—O4—K1A^xiv^	97.46 (19)
O2—K1B—O2^vi^	80.6 (4)	Fe1^xvii^—O4—K1A^xiv^	97.46 (19)
O1^iv^—K1B—O2^vi^	127.7 (6)	K1B—O4—K1A^xiv^	111.5 (3)
O2^iv^—K1B—O2^vi^	174.4 (5)	P2—O4—K1A	93.21 (17)
O4^vii^—K1B—O2^vi^	92.5 (3)	Ni1^xvii^—O4—K1A	115.51 (15)
P2—K1B—O2^vi^	73.2 (3)	Fe1^xvii^—O4—K1A	115.51 (15)
P1^viii^—K1B—O2^vi^	86.0 (4)	K1B—O4—K1A	3.5 (3)
O5^iv^—K1B—O1^vii^	93.5 (4)	K1A^xiv^—O4—K1A	113.54 (12)
O4—K1B—O1^vii^	150.5 (5)	P2—O4—K1B^xiv^	105.2 (3)
O5^vi^—K1B—O1^vii^	89.6 (4)	Ni1^xvii^—O4—K1B^xiv^	93.5 (3)
O3^vi^—K1B—O1^vii^	95.5 (4)	Fe1^xvii^—O4—K1B^xiv^	93.5 (3)
O2—K1B—O1^vii^	99.1 (5)	K1B—O4—K1B^xiv^	112.9 (2)
O1^iv^—K1B—O1^vii^	95.8 (4)	K1A^xiv^—O4—K1B^xiv^	4.0 (3)
O2^iv^—K1B—O1^vii^	48.2 (3)	K1A—O4—K1B^xiv^	115.1 (3)
O4^vii^—K1B—O1^vii^	44.5 (2)	P1—O5—Fe1	144.88 (18)
P2—K1B—O1^vii^	123.2 (5)	P1—O5—K1B^iv^	100.3 (3)
P1^viii^—K1B—O1^vii^	89.8 (4)	Fe1—O5—K1B^iv^	97.7 (3)
O2^vi^—K1B—O1^vii^	135.7 (4)	P1—O5—K1A^iv^	98.87 (15)
O5^ix^—P1—O5	108.79 (10)	Fe1—O5—K1A^iv^	102.05 (18)
O5^ix^—P1—O5^x^	110.8 (2)	K1B^iv^—O5—K1A^iv^	6.5 (2)
O5—P1—O5^x^	108.79 (10)	P1—O5—K1B^v^	95.5 (3)
O5^ix^—P1—O5^xi^	108.79 (10)	Fe1—O5—K1B^v^	101.9 (3)
O5—P1—O5^xi^	110.8 (2)	K1B^iv^—O5—K1B^v^	118.67 (12)
O5^x^—P1—O5^xi^	108.79 (10)	K1A^iv^—O5—K1B^v^	112.8 (3)
O5^ix^—P1—K1B^xii^	56.8 (2)	P1—O5—K1A^v^	96.61 (14)
O5—P1—K1B^xii^	130.8 (3)	Fe1—O5—K1A^v^	97.63 (18)
O5^x^—P1—K1B^xii^	120.3 (3)	K1B^iv^—O5—K1A^v^	124.5 (3)
O5^xi^—P1—K1B^xii^	52.3 (2)	K1A^iv^—O5—K1A^v^	118.73 (10)
O5^ix^—P1—K1B^iv^	120.3 (3)	K1B^v^—O5—K1A^v^	6.5 (2)
O5—P1—K1B^iv^	52.3 (2)		

Symmetry codes: (i) −*x*+1/2, *y*+1/2, −*z*+1/2; (ii) *x*−1/2, −*y*+3/2, −*z*+1/2; (iii) *y*−1/2, *x*+1/2, *z*−1/2; (iv) −*x*, −*y*+1, *z*; (v) *y*, −*x*+1, −*z*+1; (vi) −*y*+1, *x*, −*z*+1; (vii) −*y*+1/2, −*x*+1/2, *z*+1/2; (viii) *x*, *y*−1, *z*; (ix) −*y*+1, *x*+1, −*z*+1; (x) *y*−1, −*x*+1, −*z*+1; (xi) −*x*, −*y*+2, *z*; (xii) *x*, *y*+1, *z*; (xiii) −*y*, *x*+1, −*z*+1; (xiv) −*y*+1/2, −*x*+1/2, *z*−1/2; (xv) *y*−1/2, *x*+1/2, *z*+1/2; (xvi) *x*+1/2, −*y*+3/2, −*z*+1/2; (xvii) −*x*+1/2, *y*−1/2, −*z*+1/2.
